# Glutathione in Brain Disorders and Aging

**DOI:** 10.3390/molecules27010324

**Published:** 2022-01-05

**Authors:** Igor Y. Iskusnykh, Anastasia A. Zakharova, Dhruba Pathak

**Affiliations:** 1 Department of Anatomy and Neurobiology, University of Tennessee Health Science Center, Memphis, TN 38163, USA; 2 Department of Medical Biochemistry, Faculty of Biomedicine, Pirogov Russian National Research Medical University, Ostrovitianov St. 1, 117997 Moscow, Russia; anzakh191@gmail.com; 3 Department of Psychology, Temple University, Philadelphia, PA 19122, USA

**Keywords:** glutathione, brain, aging, disorders, neuron

## Abstract

Glutathione is a remarkably functional molecule with diverse features, which include being an antioxidant, a regulator of DNA synthesis and repair, a protector of thiol groups in proteins, a stabilizer of cell membranes, and a detoxifier of xenobiotics. Glutathione exists in two states—oxidized and reduced. Under normal physiological conditions of cellular homeostasis, glutathione remains primarily in its reduced form. However, many metabolic pathways involve oxidization of glutathione, resulting in an imbalance in cellular homeostasis. Impairment of glutathione function in the brain is linked to loss of neurons during the aging process or as the result of neurological diseases such as Huntington’s disease, Parkinson’s disease, stroke, and Alzheimer’s disease. The exact mechanisms through which glutathione regulates brain metabolism are not well understood. In this review, we will highlight the common signaling cascades that regulate glutathione in neurons and glia, its functions as a neuronal regulator in homeostasis and metabolism, and finally a mechanistic recapitulation of glutathione signaling. Together, these will put glutathione’s role in normal aging and neurological disorders development into perspective.

## 1. Introduction

Glutathione (GSH) is a tripeptide that contains cysteine, glutamic acid, and glycine residues, which are distributed ubiquitously in every cell [1]. It serves as an endogenous antioxidant that affects many cellular functions. GSH and several enzymes combine to form the glutathione system, which plays a crucial role in the utilization and regulation of reactive oxygen and nitrogen species (ROS and RNS, respectively) in organisms. Intracellular levels of GSH are maintained by direct uptake of exogenous GSH, de novo GSH synthesis, and GSH redox cycling. De novo synthesis of GSH from cysteine and glutamic acid involves catalysis by glutamate-cysteine ligase (GCL) to form gamma-glutamyl cysteine (g-GC) and the subsequent addition of glycine to g-GC by glutathione synthase (GS) [2]. During the process of GSH redox cycling, the enzyme glutathione peroxidase (GPx) oxidizes GSH to glutathione disulfide (GSSG) during detoxification of hydrogen peroxide (H_2_O_2_) or other organic hydroperoxides. The oxidized form, GSSG, can be converted back to GSH by glutathione reductase (GR). Conjugation of GSH by the enzyme glutathione S-transferase (GST) to xenobiotic compounds yields nontoxic products, thereby effecting their detoxification [3,4].

GSH serves many pivotal functions in the central nervous system, including the modulation of cellular differentiation and proliferation, apoptosis, enzyme activation, metal transport in cells, neurotransmission, and as a source of cysteine during protein synthesis (Table 1) [5,6].

GSH is also important for the early period of embryogenesis, especially during fertilization [43]. The basal level of GSH in a mouse oocyte before fertilization is high, approximately 7 mM, which is required for normal fertilization. Inhibition of GSH biosynthesis in the ovaries results in a statistically significant decrease of 90% in GSH levels in oocytes, which prevents nuclear de-condensation in the sperm. After successful fertilization, the zygote undergoes a series of cell divisions to produce daughter cells. During this process, the reduction of the cytoplasm, as cells divide, results in a concomitant drop in GSH levels. For example, although a dividing embryo at the blastocyst stage has an overall volume that is similar to that of an unfertilized oocyte, its GSH level drops to approximately 0.12 pmol, compared to 1.2 pmol in an oocyte. This process continues for approximately 4–5 days, until the embryo is transferred from the oviduct to the uterus, when GSH levels decline to baseline levels [43].

**Figure 1:**
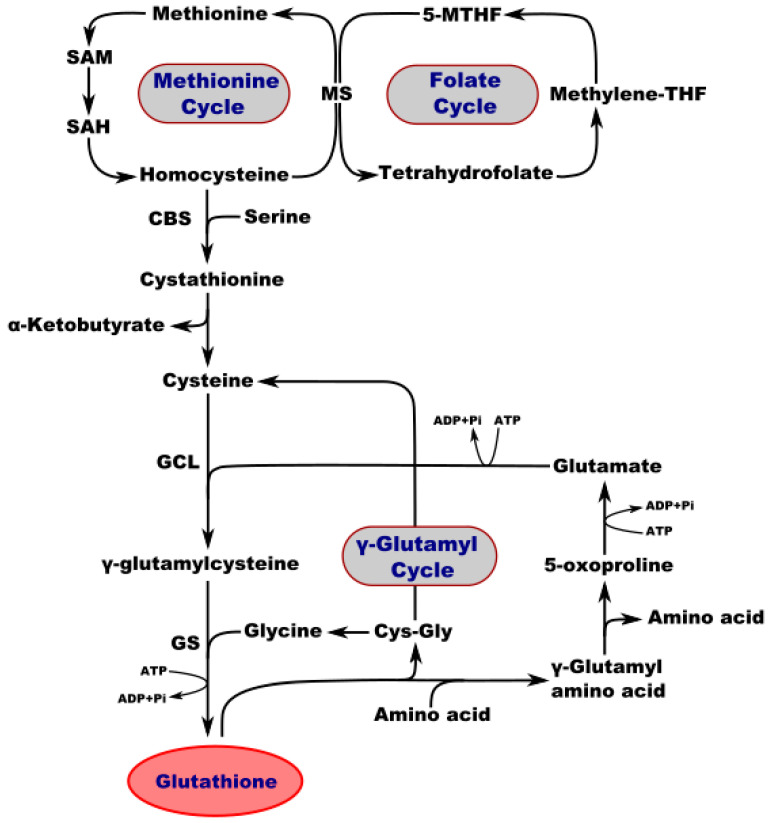
Interconnection of metabolic and synthetic pathways of GSH. SAM—S-adenosylmethionine; SA—S-adenosylhomocysteine; MS—methionine synthase; 5-MTHF—5-methyltetrahydrofolate; Methylene-THF—methylene tetrahydrofolate; CBS—cystathionine b-synthase; GCL—glutamate-cysteine ligase; GS—GSH synthetase; CysGly—cysteine-glycine.

Interestingly, the concentration of GSH in different tissues of adult mammals may vary from 1 to 10 mM [44]. It is present at high levels in the brain, with a total GSH content of 0.5–3.4 µmol/g [45]. In the mammalian central nervous system (CNS), the highest concentration of GSH is found in glial cells of the cortex. GSH absorption is the highest in the retina as compared to other brain regions, including the hypothalamus, striatum, spinal cord, midbrain, medulla, pons, hippocampus, cerebellum, and cerebral cortex [46]. The brain and other tissues use a common pathway for GSH synthesis. Consecutive reactions of two enzymes are required to synthesize GSH. First, γ-glutamylcysteine synthetase uses glutamic acid and cysteine as substrates to generate glutamylcysteine (γGluCys). Glutathione synthetase then produces GSH from glycine and γGluCys (Figure 1). However, detoxification of compounds by GSH coupling occurs primarily in the kidneys and liver, but not in the brain. GSH-S-alkyl transferase and GSH-S-transferase enzymes in rat brains exhibit little activity, while the cerebellum and brainstem exhibit a low level of GSH-S-arene-oxide transferase activity. The majority of GSH (97%) in the brain is in its reduced form. In vivo, brain ischemia induces a decrease in GSH levels, but this is not accompanied by any reciprocal increase in GSSG [45,47,48].

**Figure 2:**
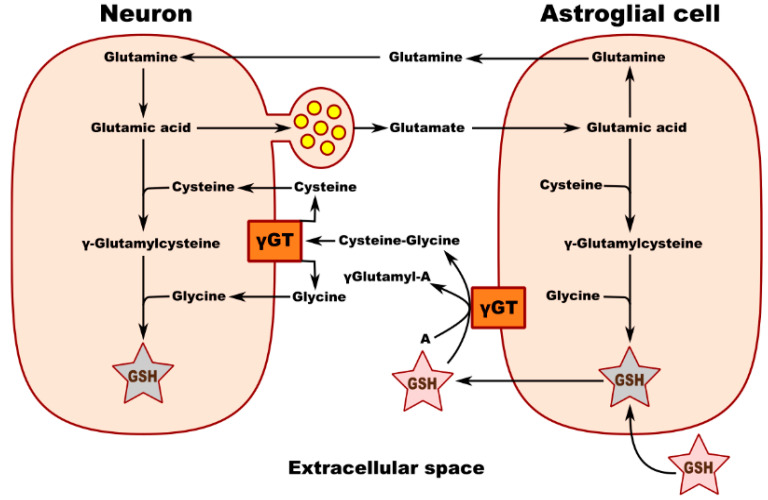
Schematic of neuronal GSH transport and metabolic interaction between neurons and astroglial cells. γ GT—gamma-glutamyl transpeptidase; A—acceptor of g-glutamyl moiety transferred from GSH by γ GT.

Metabolic interaction between neurons and astrocytes is critical for glutathione synthesis. An in vitro experiment performed in co-cultured neurons and astrocytes showed that GSH levels in neurons increase in the presence of astrocytes, most likely due to the transfer of a cysteine precursor from astrocytes to neurons, thereby upregulating GSH synthesis in the recipient neurons. Membrane-associated gamma-glutamyl transpeptidase (γGT) on the surface of astrocytes converts extracellular GSH to the dipeptide CysGly and subsequently to Cys and Gly, which are transported into the neuron, where they serve as precursors for the synthesis of neuronal GSH (Figure 1). Inhibition of the γGT reaction completely prevents this astroglia-induced increase in the neuronal GSH level. Figure 2 shows the theoretical metabolic interaction of astrocytes and neurons in terms of glutathione metabolism [47].

Many cell types and tissues release GSH into the circulatory system, which promotes its transfer between cells. In the CNS, GSH is present in both the extracellular fluid and in the cerebrospinal fluid (CSF) [48]. The plasma levels of total glutathione (GSH + GSSG) in rats is 22–27 mM GSH equivalent, as measured by the glutathione reductase recycling method. GSH represents about 85% of the total glutathione [49]. Several organs including the brain absorb GSH from the plasma, either by direct uptake of GSH by carrier-mediated transport, or by breakdown of GSH by γ GT and dipeptidases and subsequent transport of Glu, Gly, and Cys amino acids [50]. Although there is a specific transport system for cysteine, it competes with other plasma amino acids for L-system transport across the blood–brain barrier [51,52].

The purpose of this review is to outline the vital role of GSH in the brain, particularly in neurodegenerative diseases and aging. The general introduction related GSH metabolism to the extended role of this important antioxidant in the central nervous system (CNS). The remainder of the review will provide some specific mechanistic explanations regarding the role played by GSH in development and age-related changes in the CNS.

## 2. A Pivotal Role for GSH in the Regulation of Homeostasis and Metabolism in the Nervous System

During the aging process, GSH plays a vital role in neuronal defense against damage caused by oxidants like ROS and RNS. Various neurodegenerative disorders are characterized by the depletion of cellular GSH, probably due to its counteracting oxidative stress and calcium ion (Ca^2+^) imbalance [53]. There is marked heterogeneity in the cellular distribution of GSH in the CNS neurons of adult rats, gerbils, and rabbits. The non-neuronal elements of the CNS and the peripheral nervous system (PNS), namely the glia, ependyma, and endothelia, exhibit high levels of GSH not found in the neuronal cell bodies or granule cells. GSTs are also likely to be heterogeneously distributed in the CNS. For example, astrocytes and oligodendrocytes express µ-GST and n-GST, respectively, while pial, ependymal, and vascular elements express various quantities of both µ- and n-GST. Alpha-GST is present in both neurons and non-neuronal elements of the nervous system. The developing nervous system is exposed to various xenobiotics through the placental circulation and undergoes a dramatic change in the oxygen environment at birth [54]. Therefore, the GSH system plays an important role in the regulation of redox homeostasis and protects newborns against the hyperoxic extrauterine environment.

Mitochondria are an important source of ROS and RNS. Roughly 10 to 20% of GSH is contained in the mitochondria in neural cells and most other tissues [55]. The mitochondrial compartment contains more GSH than any other cellular compartment, yet the mitochondria do not contain the enzymes necessary for its biosynthesis [56]. Instead, mitochondria import GSH from the cytosol effectively using specific GSH transport systems [57]. The exact system used in each case depends on the substrate and the tissue. For example, the import of GSH into kidney and liver mitochondria can occur primarily using 2-oxoglutarate (2-OG; SLC25A11) or dicarboxylate (DIC; SLC25A10) as a carrier, while tricarboxylate (TTC, SLC25A1) serves as a carrier in CNS neurons and astrocytes. The 2-OG carrier (OGC) mediates the exchange of cytosolic GSH and mitochondrial dicarboxylates, including 2-OG [57].

The tripeptide structure of GSH suggest its potential role as a neuroactive molecule. All three of its amino acid residues can interfere with neuronal signaling through glutamate (Glu) receptors. Loss or improper function of Glu receptors or alteration of cerebral GSH levels can lead to neuropsychiatric symptoms or neurological abnormalities. The conformational flexibility of GSH allows for its binding to all classes of Glu receptors via its glutamyl residue due to its similarity to the natural receptor agonist, L-glutamate. The cysteine residue in GSH has neurotoxic properties in its free state, although it is not toxic in peptide form. At low concentrations, GSH is neuroprotective, but at high (millimolar; mM) concentrations, GSH may affect the redox state of glutamate receptors via its free thiol group. Similar to free glycine, the glycine residue in GSH serves as a co-agonist of the N-methyl-D-aspartate (NMDA) receptor and as the main inhibitory neurotransmitter in the spinal cord [58,59]. Alterations in NMDA receptor function can alter the calcium signaling cascade and affect synaptic plasticity, leading to pathophysiological changes in the CNS.

The redox potential regulates the activity of the NMDA receptor, which normally fluctuates between fully oxidized and fully reduced states [60,61,62,63]. Redox homeostasis in the brain is very important, as the consumption of high levels of oxygen produces many harmful free radicals, including superoxide anions (O_2_^•−^), hydroxyl radicals (^•^OH), lipoperoxide radicals (LOO·), nitric oxide radicals (NO^•^), and nitrogen dioxide radicals (NO_2_^•^) [5,64]. As an antioxidant, the GSH system maintains thiols by the scavenging of free radicals and by reversible thiol-disulfide exchange reactions. Therefore, it is a putative endogenous redox modulator of the NMDA receptor activity [60,61,62,63].

Mitochondrial GSH serves as a natural antioxidant store. Selective depletion of mitochondrial but not cytoplasmic GSH from cerebellar granule neurons (CGNs) is associated with enhanced permeability of mitochondrial transition pores, increased ROS production, and increased cell death. Interestingly, different cell types in the CNS exhibit varied sensitivity to oxidative stress due to their differing levels of mitochondrial GSH [65]. For example, astrocytes are more vulnerable to oxidative and nitrosative stress than cells with higher levels of GSH [65]. Although the human brain constitutes only 2% of the total body weight, it utilizes 20% of the oxygen consumed by the body. Neurons support high levels of oxidative phosphorylation, which is expected to generate a large amount of ROS. Cells that generate such high levels of ROS require an antioxidative defense system to protect cellular structures from ROS damage [48,66]. In brain cells, the antioxidant function of GSH plays a key role in their defense against oxidative stress.

Depletion of this cellular reservoir of GSH leads to the amplification of oxidative and nitrosative cell damage, hypernitrosylation, increased levels of inflammation, disturbances in intracellular signaling pathways for p53, Janus kinases (JAK), and nuclear factor-κB (NF-κB); decreased DNA synthesis and cell proliferation; activation of cytochrome c, inactivation of complex I of the electron transport chain and the apoptotic pathways; blockage of the methionine cycle; and compromised epigenetic regulation of gene expression. GSH depletion also has deleterious effects on redox homeostasis of the immune system, molecular pathways involved in oxidative and nitrosative stresses, control of energy production, and mitochondrial survival in different cell types [6]. GSH imbalance and/or deficiency in the neurons is involved in the pathogenesis of brain disorders, including AD, amyotrophic lateral sclerosis (ALS), autism, bipolar disorder, Huntington’s disease (HD), multiple sclerosis (MS), Parkinson’s disease (PD), and schizophrenia [5]. Many neurological disorders are associated with impaired balance between ROS generation and activity of the antioxidant system, particularly GSH, as reviewed recently [47]. For example, impaired GSH metabolism contributes to PD pathogenesis. Abnormal mitochondrial functioning may also play a crucial role in these diseases [47].

Congenital GSH synthetase deficiency can cause mental retardation, spastic quadriplegia (also called spastic tetraparesis; a form of cerebral palsy), cerebellar dysfunction, and γ-glutamyl-cysteine synthetase deficiency, which can lead to spinocerebral degradation, ataxia, and the absence of lower-limb reflexes [67,68,69,70]. The concentration of GSH decreases in the substantia nigra, during both ontogenesis and in the degenerating dopaminergic neurons of PD patients [71,72,73,74]. GSH levels are reduced in the brains of epilepsy patients during convulsive seizures [75] and in genetically epileptic (tg/tg) transgenic mice [76]. Furthermore, the depletion of GSH in adult rats by treatment with buthionine sulfoximine leads to the development of seizures [77].

Thus, glutathione plays an important role in the onset and progression of neurological disorders and neurodegenerative diseases, and may serve as a biomarker for diagnostic screening for these disorders.

## 3. Glutathione Regulates Aging and Neurodegeneration

**Figure 3:**
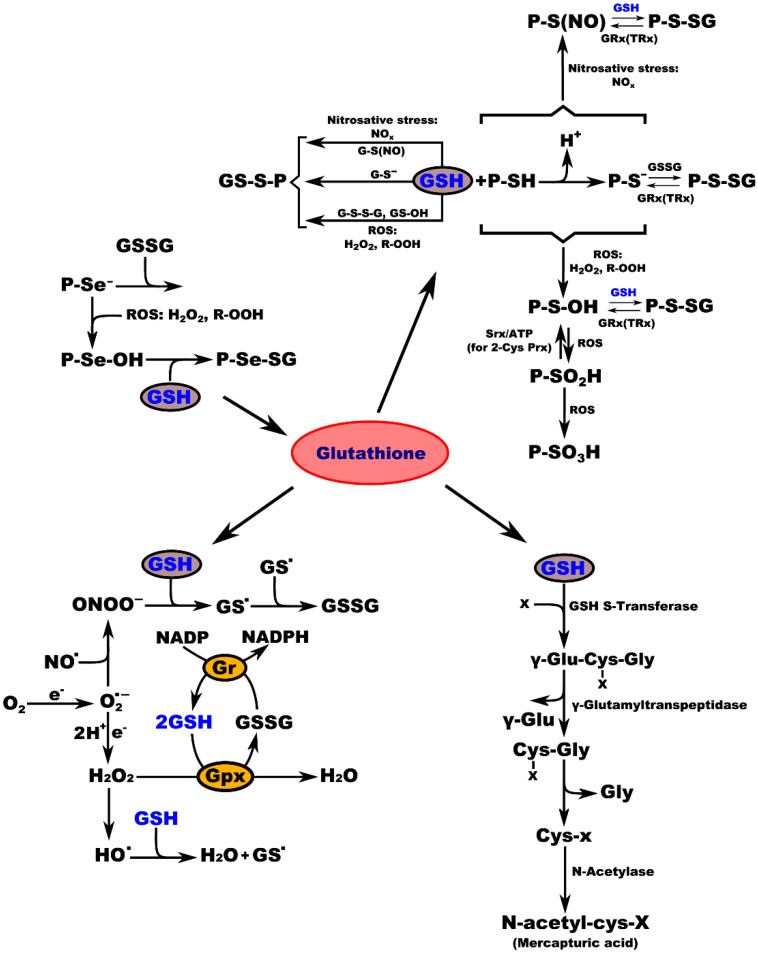
Interconnection of GSH metabolic pathways. P-protein; P-Se^−^-protein-selenate anion; ROS-reactive oxygen species; H_2_O_2_-hydrogen peroxide; R-OOH-hydroperoxide; P-Se-OH-protein-selenenic acid; GSH-glutathione, P-Se-SG-protein-selenate; NO_x_-reactive nitrogen species; G-S(NO)-S-nitroso-glutathione; G-S^-^-glutathione-thiolate; GSSG-glutathione disulfide (oxidized glutathione); GS-OH^-^-glutathione sulfenic acid; P-SH-protein sulfhydryl group; H^+^-proton; P-S^−^-protein-thiolate anion; P-S(NO)-protein-S-nitroso thiol; P-S-SG-protein-S-glutathione; GRx(TRx)-glutaredoxin (thioredoxin); P-S-OH-protein-sulfenic acid; Srx-sulfiredoxin; ATP-adenosine triphosphate; 2-Cys Prx-2-cysteine peroxiredoxin; P-SO_2_H-protein-sulfinic acid; P-SO_3_H-protein-sulfonic acid; O_2_-oxygen; e**^.^**-electron radical; O_2_^−^-superoxide radical; NO-nitric oxide radical; ONOO^−^-peroxynitrite; GS-glutathione radical; e^−^-electron; Gr-glutathione reductase; GPx-glutathione peroxidase; NADP-nicotinamide adenine dinucleotide; NADPH-nicotinamide adenine dinucleotide phosphate; H_2_O-water; HO^−^-hydroxyl radical; x-compound with an electrophilic center that can be conjugated to GSH by GSH S-transferase; γ-Glu-Cys-x-Gly-γ-glutamate-cysteine-x-glycine; γ-Glu-γ-glutamic acid; Cys-x-Gly-cysteine-x-glycine; Gly-glycine; Cys-x-cysteine-x; N-acetyl-cys-x-mercapturic acid-x.

Biochemical studies of the effects of aging on the antioxidant systems highlighted the existence of age-related changes in the GSH content of the nervous system. Aging is often associated with impairments in CNS function, resulting from loss of neurons and leading to diminished cognitive performance. Free radicals, particularly oxygen radicals, play important roles in age-related changes and in the pathogenesis of several neurodegenerative diseases. Our defense against oxygen radicals relies on the enzymes of the glutathione/thioredoxin antioxidant system, which inactivate ROS and NOS, glutathione peroxidase detoxifies peroxides, including H_2_O_2_ and peroxides that are generated during the oxidation of membrane lipids. The enzymatic oxidation of GSH to the disulfide GSSG determines the extent of the reduction in the peroxide levels. To maintain the cellular balance of GSH and GSSG, GSH can be regenerated from GSSG by glutathione reductase with NADPH as a cofactor [57]. GSH provides approximately 90% of the non-protein sulfhydryl groups in the cells and maintains the thiol status of the cellular proteins (Figure 3) [78].

In rats, brain GSH levels that are already high in prenatal animals increase by approximately 20% during the first year of postnatal life and decline thereafter [54]. GSH associates with molecules and signaling pathways that regulate iron metabolism, apoptosis, and thiol-redox control. Although many conditions are associated with low GSH levels (AD, autism, PD, MS, and schizophrenia, as noted above and in Table 2, a connection to a decrease of glutamate-cysteine ligase (GCL) activity or loss of GCL expression has not been demonstrated for all of them (Table 2) [5,79]. In relation to this, study of GCL subunit polymorphism is necessary.

Polymorphisms of GCLM are associated with a decrease in GCLC expression in hemolytic anemia patients [80], both a decrease in GSH levels and a subsequent onset of drug resistance in cancer cell lines [81], and both reduced GCL activity and correspondingly decreased GSH levels in schizophrenia patients [82,83]. GCLM polymorphisms are also associated with NO-mediated impairment of coronary vasomotor function and an increased risk of myocardial infarction (heart attack). More studies are needed to determine whether these polymorphisms also affect the pathology of other diseases.

Some health conditions are associated with a change in the activity and/or expression of GCL that are unrelated to known polymorphisms. Recent studies in rodents revealed the effect of aging on GSH homeostasis in different tissues. In particular, they noted decreased levels of GSH with age in all tissues tested, which was associated with a reduced expression of GS and GCL in the absence of any changes in the expression of GGT and GSH reductase, cysteine availability, or the amount of GSSG as an indicator of oxidative stress [83]. Aging in individuals of both sexes is accompanied by decreased GSH levels. However, the reduction of GSH and GCL mRNA levels was more pronounced in most tissues from the male mice, compared to those from the female mice. Exogenous estrogen enhances the expression of GCL and GS enzymes and GSH levels in the livers of male and female mice, but not in the heart or the brain [83].

Glutathione synthase deficiency (GSSD) is a very rare autosomal-recessive genetic disorder that has been described in only approximately 70 patients world-wide. The disease is characterized by different levels of hemolytic anemia, metabolic acidosis, retinal dystrophies, susceptibility to bacterial infections (including sepsis), progressive cerebral and cerebellar degeneration, psychomotor and intellectual retardation, and other neurological symptoms [84]. Thus, an age-dependent decrease in GSH levels could contribute to the onset of many age-related diseases, but exactly how normal aging influences the expression of GS and GCL at a molecular level remains unclear [79,83]. Interestingly, because red blood cells contain higher levels of GSH compared to other cells in the body, the red blood cells of GSSD patients are more susceptible to the negative effects of oxidative stress, which often leads to hemolytic anemia. Patients with severe GSSD also exhibit neurological findings, which include motor disturbances and developmental delays [85]. Recent studies have described the role of glutathione in a-Synucleinopathies and Tauopathies [86,87]. Being able to trans-synaptically spread from neuron to neuron as a “seed” form, aSyn or tau proteins exist in multiple aggregate conformations that are, most likely, responsible for the clinical heterogeneity of a-Synucleinopathies and Tauopathies [88,89,90,91,92,93]. Interestingly, Esteban Luna et al. showed that toxicity resulting from PFF-seeded aSyn pathology could be attenuated by N-acetyl-cysteine through a glutathione-dependent process. Thus, it was hypothesized that PFF-induced toxicity is, at least to a certain degree, mediated by glutathione depletion, which becomes critical when the cell accumulates an extensive amount of aggregates [86]. A strong association of the neurodegenerative diseases with the depletion of the GSH system has also been demonstrated in other studies [94,95]. Depletion of antioxidant systems in neurons, especially the glutathione system, leads to an increase in neuroinflammation and has an important role in the pathogenesis of neurodegenerative disorders [94,95,96]. The development and progression of ALS due to an impairment of the GSH system-medicated antioxidant protection leads to ALS-induced neuronal toxicity [96]. Parkinson’s disease-related neurodegeneration and neuroinflammation also are known to be related to the depletion of the GSH system [94].

These data suggest that the genetic features of specific enzymes of the glutathione antioxidant system and their control of oxidative and nitrosative stresses throughout the lifespan highlight their likely involvement in defining the fate of normal aging, particularly in the CNS. Thus, the study of the glutathione system will remain one of the most pressing research areas in the biology of brain aging for many decades to come.

## Figures and Tables

**Table 1:** Physiological and metabolic functions of GSH.

Function	Role of GSH	Reference
Enhancement of immune system function	Protects against inflammatory pathologies Impaired immunological function caused by cysteine and glutathione deficiency is restored when supplemented with cysteine	[7,8]
Prevention of oxidative cell damage	Serves as an antioxidant	[9,10]
Prostaglandin synthesis	Inhibits prostaglandin synthesis at elevated levels	[11]
Transport of metals across membranes	Chelates reactive metals and facilitates their transport across cell membranes	[12]
Transfers metals between ligands	Forms coordinate-covalent adducts with several transition metals and transports them between ligands	[13]
Protein synthesis	Is involved in post-translational modification of proteins	[14]
DNA synthesis and repair	Efficient scavenging of OH^•^ and secondary radicals; participates in rejoining of X-ray induced DNA strand breaks; activates T-cell proliferation; provides a source of cysteine moieties	[15,16,17]
Amino acid transport	Participates in amino acid permeation system; assists with transmembrane transport of amino acids by acting as a donor of g-glutamyl groups to amino acids, catalyzed by membrane-bound g-glutamyl transpeptidase	[18,19]
Enzyme activation	As a reducing agent, is important for activation of peptidyl-arginine deiminase; binds to microsomal prostaglandin E synthase type 2 (mPGES2), a heme group is attached to GSH via an iron-sulfur bond, and the resulting enzyme catalyzes degradation of PGH2	[20,21]
Metabolism of toxins and carcinogens	Detoxification of electrophilic xenobiotics and endogenous compounds after spontaneous or enzymatic (GSH-S-transferase) GSH conjugation; conjugation of 1,2-dibromoethane	[22,23]
Redox reactions	Neutralizes charges on ROS, RNS, and other reactive species	[4]
Source of cysteine	Serves as an extracellular source of cysteine	[24]
Metal Homeostasis	Reduction of Cr^6+^	[25,26]
NMDAR responses, neuronal activity	Enhances NMDAR responses and neuronal activation by calcium or electrical signals, while its depletion or oxidation results in NMDAR hypofunction	[27]
Calcium signaling	Participates in calcium signaling (mM range), especially in Müller glia; can regulate GABA release with protective effects on the retinal neuron-glial circuit; modulates influx of Ca^2+^ and oxidative toxicity through the TRPM2 channel in rat dorsal root ganglion neurons	[28,29]
Myelin maturation	Its deficit impairs maturation of myelin	[30]
Neuro-modulator, neurotransmitter	Has binding sites for a putative receptor, likely the glutamate receptor; can serve as a neuromodulator or neurotransmitter	[31]
Source of neuronal glutamate	Serves as a physiologic reservoir of neuronal glutamate	[32]
Neuronal differentiation	Induces neuronal differentiation in rat bone marrow stromal cells	[33]
Apoptosis	Intracellular levels decrease after activation of the mitochondrial death receptor, drug exposure, or oxidative stress	[3]
JAK1-STAT3 signaling	Reducing its intracellular pool enhances LIF-induced JAK1-STAT3 signaling	[34]
cAMP signaling	Upregulates cAMP signaling via G protein alpha 2	[35]
T-cell metabolism	Primes T cell metabolism for inflammation	[36]
Activates MAPK pathways	Reducing its intracellular pool activates mitogen-activated protein kinase (MAPK) pathways	[37]
NMDA-R agonist	Act as an agonist of the NMDA receptor	[27,38]
Ca^2+^-activated K^+^ channels	Reduced form increases channel activities, while oxidized form inhibits channel activities	[39]
Mammalian development	Essential for embryonic development, as knockout of homozygous glutathione synthetase is lethal in mice; implicated in maintenance of meiotic spindle morphology in oocytes	[40,41]
Prevention of motor neuron degeneration	Decreased levels promote degeneration of motor neurons in vitro and in vivo	[42]

**Table 2:** Activity of glutathione-related enzymes and content of glutathione in the blood and brain of patients with different brain diseases (5).

Disease/Disorder	Blood	Brain
Alzheimer’s disease (AD)	Decreased GSH	Decreased GSH
	Decreased GSH/GSSG	Decreased GST
	Increased GSSG	-
	Decreased Gpx activity	-
Amyotrophic lateral sclerosis (ALS)	Decreased GSH (erythrocytes)	Decreased GSH (motor cortex)
	-	Decreased GST (motor cortex)
Autism	Decreased GSH	Decreased GSH in (celebellum and temporal cortex)
	Increased GSSG	Decreased Gpx activity
	Decreased Gpx activity (erythrocytes)	Decreased GST activity
	Decreased GST activity (erythrocytes)	Decreased GCL activity
	Decreased GSH/GSSG	Decreased GSH/GSSG (cerebellum and temporal cortex)
Bipolar disorder	Decreased GSH	Decreased GSH (hippocampus and anterior cortex)
	Increased GSSG	-
	Decreased GSH/GSSG	-
Huntington’s disease (HD)	Decreased GSH	-
Multiple sclerosis	Decreased GSH	Decreased GSH
Parkinson’s disease (PD)	Decreased GSH	Decreased GSH
	-	Increased Gpx protein
	-	Increased GST protein
Schizophrenia	Decreased GSH	Decreased GSH
	Decreased GSH/GSSG	-
	Increased GSSG	-

## Data Availability

Not applicable.

## Sample Availability

Samples of the compounds are not available from the authors.

## Abbreviations

GSSG	oxidized glutathione
GSH	reduced glutathione
ROS	reactive oxygen species
H_2_O_2_	hydrogen peroxide
P-SH	protein sulfhydryl group
P-S-SG	protein-S-glutathione
GRx	glutaredoxin
TRx	thioredoxin
P-S-OH	protein-sulfenic acid
Srx	sulfiredoxin
ATP	adenosine triphosphate
NO	nitric oxide radical
ONOO^−^	peroxynitrite
GS^.^	glutathione radical
Gr	glutathione reductase
GPx	glutathione peroxidase
NADP	nicotinamide adenine dinucleotide
NADPH	nicotinamide adenine dinucleotide phosphate
PFF	pre-formed fibrils
